# Superior performance in classification of breast cancer molecular subtype and histological factors by radiomics based on ultrafast MRI over standard MRI: evidence from a prospective study

**DOI:** 10.1007/s11547-025-01956-6

**Published:** 2025-01-25

**Authors:** Juhyun Jeong, Sungwon Ham, Bo Kyoung Seo, Jeong Taek Lee, Shuncong Wang, Min Sun Bae, Kyu Ran Cho, Ok Hee Woo, Sung Eun Song, Hangseok Choi

**Affiliations:** 1 Department of Radiology, Korea University Ansan Hospital, Korea University College of Medicine, 123 Jeokgeum-Ro, Danwon-Gu, Ansan City, 15355 Gyeonggi-Do Korea; 2 Healthcare Readiness Institute for Unified Korea, Korea University Ansan Hospital, Korea University College of Medicine, Ansan, Republic of Korea; 3 Department of Radiology and Nuclear Medicine, Amsterdam University Medical Center, Amsterdam, The Netherlands; 4 Department of Radiology, Korea University Anam Hospital, Korea University College of Medicine, Seoul, Republic of Korea; 5 Department of Radiology, Korea University Guro Hospital, Korea University College of Medicine, Seoul, Republic of Korea; 6 Medical Science Research Center, Korea University College of Medicine, Seoul, Republic of Korea

**Keywords:** Breast cancer, Radiomics, Magnetic resonance imaging, Histological factor, Subtype

## Abstract

**Purpose:**

To compare the performance of ultrafast MRI with standard MRI in classifying histological factors and subtypes of invasive breast cancer among radiologists with varying experience.

**Methods:**

From October 2021 to November 2022, this prospective study enrolled 225 participants with 233 breast cancers before treatment (NCT06104189 at clinicaltrials.gov). Tumor segmentation on MRI was performed independently by two readers (R1, dedicated breast radiologist; R2, radiology resident). We extracted 1618 radiomic features and four kinetic features from ultrafast and standard images, respectively. Logistic regression algorithms were adopted for prediction modeling, following feature selection by the least absolute shrinkage and selection operator. The performance of predicting histological factors and subtypes was evaluated using the area under the receiver-operating characteristic curve (AUC). Performance differences between MRI methods and radiologists were assessed using the DeLong test.

**Results:**

Ultrafast MRI outperformed standard MRI in predicting HER2 status (AUCs [95% CI] of ultrafast MRI vs standard MRI; 0.87 [0.83–0.91] vs 0.77 [0.64–0.90] for R1 and 0.88 [0.83–0.91] vs 0.77 [0.69–0.84] for R2) (all *P* < 0.05). Both ultrafast MRI and standard MRI showed comparable performance in predicting hormone receptors. Ultrafast MRI exhibited superior performance to standard MRI in classifying subtypes. The classification of the luminal subtype for both readers, the HER2-overexpressed subtype for R2, and the triple-negative subtype for R1 was significantly better with ultrafast MRI (*P* < 0.05).

**Conclusion:**

Ultrafast MRI-based radiomics holds promise as a noninvasive imaging biomarker for classifying hormone receptors, HER2 status, and molecular subtypes compared to standard MRI, regardless of radiologist experience.

**Supplementary Information:**

The online version contains supplementary material available at 10.1007/s11547-025-01956-6.

## Introduction

Standard dynamic contrast-enhanced magnetic resonance imaging (MRI) can be time-consuming, requiring approximately 5–7 min after contrast agent injection and review of approximately 2500 images by radiologists [1]. However, its temporal resolution, typically greater than 60 s, often obscures important kinetic information of breast tumors in the early stage [2]. In contrast, ultrafast dynamic contrast-enhanced MRI offers extremely fast temporal resolution (< 8 s), allowing for shorter scan times and better assessment of tumor kinetics [3, 4]. Ultrafast MRI can improve lesion conspicuity by capturing cancer enhancement before background enhancement, maintaining diagnostic performance comparable to standard MRI [5, 6]. It also provides quantitative kinetic parameters such as time to enhancement (TTE), maximum slope, and wash-in slope [3, 7, 8]. Recent studies have demonstrated its effectiveness in predicting neoadjuvant chemotherapy response and histological factors and subtypes of breast cancer. The St. Gallen International Expert Consensus Panel recommends stratifying breast cancer patients for treatment based on their hormone receptor (HR) or human epidermal growth factor receptor 2 (HER2) status [9]. Breast cancer treatment includes local therapies, like surgery and radiation, and systemic therapies. According to the Panel, systemic therapy is determined by subtype: luminal (HR $+$), HER2-overexpressed (HR $-$, HER2 $+$), and triple-negative (HR $-$, HER2 $-$). Systemic options include endocrine therapy for HR-positive cancers, anti-HER2 therapy for HER2-positive cancers, and chemotherapy for HR- and HER2-negative cancers. Pathologic complete response after neoadjuvant chemotherapy is strongly correlated with subtype, particularly HER2-positive or triple-negative cancers. [10, 11]. Therefore, assessing histological factors, specifically HR and HER2, as well as molecular subtypes, is essential for treatment planning and predicting therapy response.

Radiomics, extracting high-dimensional data from radiological images, provides valuable insights into tumor biology not visible to the human eye [12]. Quantifiable features can provide systematic analysis of images by overcoming the limitations of subjective analysis and dependence on radiologists' experience in MRI interpretation [13]. According to the radiomics quality scoring system, good radiomics studies begin with high-quality image processing, prospective design, biological correlation, and individual reader evaluation [14]. One of main clinical roles of radiomics in breast cancer is tumor classification, and standard MRI has shown good performance in this role [15, 16]. Radiomics approaches for classifying histological characteristics enable a noninvasive, reproducible assessment of the entire tumor. This allows for frequent reassessment through repeated imaging tests during the course of treatment. As a result, radiomics can serve as a “virtual biopsy” [13], which is pertinent in the current era of personalized medicine.

However, only a few studies have compared the performance of ultrafast MRI and standard MRI radiomics. A pilot study by Drukker et al. [17] reported that ultrafast MRI radiomics performed comparably to standard MRI in breast cancer diagnosis. We hypothesized that radiomics classification performance using ultrafast MRI would be similar to that using standard MRI. This prospective study aims to demonstrate the prediction performance in ultrafast MRI-derived radiomics and to compare the performance with that from standard MRI-derived radiomics, in determining histological factors and subtypes in invasive breast cancer. Additionally, the impact of tumor masks from radiologists with different seniority was also elaborated.

## Materials and methods

### Study participants

**Fig. 1:**
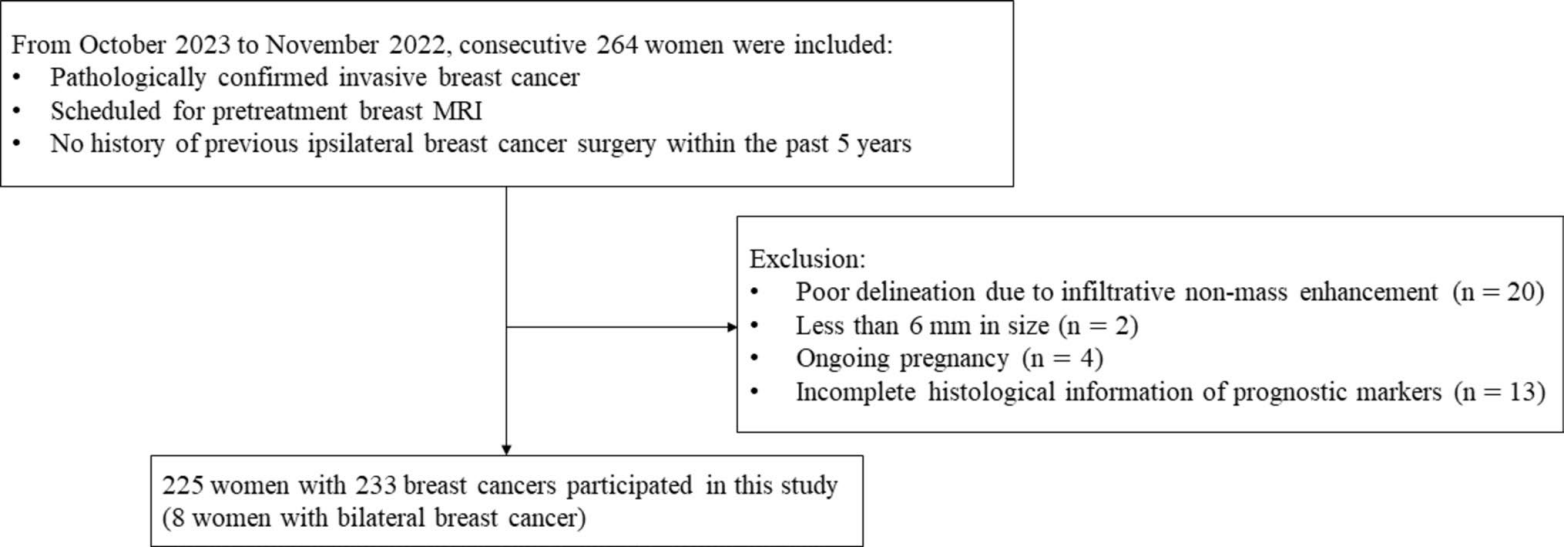
Flowchart of the study participants. A total of 233 breast cancers of 225 participants were included

This study was approved by the institutional review board, and written informed consent was obtained from all participants. This study was registered at clinicaltrials.gov (NCT06104189). Sample size determination is described in Supplementary information. From October 2021 to November 2022, consecutive 264 women with pathologically proven invasive breast cancer and scheduled pretreatment breast MRI were enrolled. The inclusion criteria were: (a) participants diagnosed pathologically with invasive breast cancers with core needle biopsy, not excision or vacuum-assisted biopsy; and (b) participants without previous ipsilateral breast surgery within the last 5 years. Thirty-nine women were excluded for the following reasons: (a) poor cancer delineation due to infiltrative enhancement or inflammatory cancer (*n* = 20); (b) less than 6 mm in size (*n* = 2); (c) ongoing pregnancy (*n* = 4); or (d) incomplete histological information (*n* = 13). Eight participants presented with concurrent bilateral breast cancer, and each lesion was examined as an individual lesion. Therefore, 233 breast cancers from 225 participants were included for analysis (Fig. 1).

### MRI protocol and feature evaluation

**Fig. 2:**
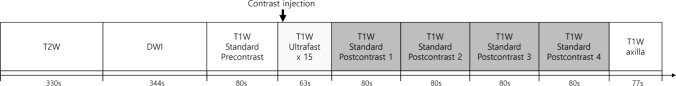
Full MRI protocol used in our study. *T1* T1-weighted, *T2* T2-weighted, *DWI* diffusion-weighted imaging

Breast MRI was performed using a 3.0 T scanner (Ingenia Elition X; Philips Healthcare) with a dedicated 16-channel bilateral breast coil. MRI protocol is presented in Fig. 2. All images were obtained with bilateral axial views and fat saturation. The protocol included T2-weighted, diffusion-weighted, precontrast T1-weighted, ultrafast dynamic contrast-enhanced T1-weighted, standard dynamic contrast-enhanced T1-weighted, and T1-weighted axillary images. Precontrast images of standard and ultrafast MRI were first performed, followed by injection of the contrast agent, gadoterate meglumine (Uniray; Dongkook Pharmaceutical Co., Ltd), and 14 ultrafast and four standard acquisitions. The contrast agent was given at a dose of 0.1 mmol/kg at a flow rate of 1.5 mL/s, followed by a 30-mL saline flush. Four-dimensional time-resolved angiography with keyhole techniques was used for ultrafast images, which were acquired within 4.2 s per image. Consecutively, standard MRI was obtained at 58.8, 138.8, 218.8, and 298.8 s after contrast injection. We evaluated kinetic and radiomic features on standard MRI and ultrafast MRI, respectively. The detailed acquisition parameters for ultrafast and standard MRI are summarized in Table 1.

**Table 1:** Acquisition parameters for ultrafast and standard MRI

Parameter	Ultrafast MRI	Standard MRI
Sequence	Turbo field echo	eTHRIVE
Acquisition time (min)	1:03 (4.2 s × 15)	5:20 (80 s × 4)
Fat suppression	SPAIR	SPAIR
Repetition time (ms)	4.1	4.7
Echo time (ms)	2.1	2.1
Flip angle (°)	12	12
Matrix	340 × 340	340 × 340
Field of view (mm^2^)	340 × 340	340 × 340
Parallel imaging factor	SENSE (4, 1.5)	SENSE (3.1, 1)
Voxel size (mm)	1 × 1 × 1	1 × 1 × 1
Sharing method	CENTRA-Keyhole Central size (%): 30 Reference scan: first	
Slice thickness (mm)	1.0	1.0

*eTHRIVE* enhanced T1-weighted high-resolution isotropic volume examination*, SPAIR* spectral attenuated inversion recovery, *SENSE* sensitivity encoding, CENTRA contrast-enhanced timing robust angiography, *NA* not applicable

On standard MRI, four kinetic parameters were extracted using a commercially available computer-aided diagnosis (CAD) system (CADstream software version 5,4,0,190, Merge Healthcare): peak enhancement (%), washout component (%), plateau component (%), and persistent component (%). Pixels with signal intensity that increased above the 50% threshold in the initial contrast-enhanced images compared to precontrast images were shown in color [18]. Color was coded according to changes in the pixel values between the initial contrast-enhanced images and delayed contrast-enhanced images [19, 20]. Washout type was defined as a more than 10% decline in pixel signal intensity in the delayed contrast-enhanced images compared to the initial contra-enhanced images (color-coded red). Persistent type was defined as an increased pixel signal intensity of more than 10% in delayed contrast-enhanced series from the initial contrast-enhanced series (color-coded blue). Plateau type was defined as less than an 10% change in pixel signal intensity in the delayed contrast-enhanced series compared with the initial contrast-enhanced series (color-coded yellow). Based on the enhancement type of each pixel in the tumor lesion, the proportions (%) of pixels with washout, plateau, and persistent patterns within in a tumor volume were quantified. Peak enhancement was defined as the percentage increase in signal intensity of the highest pixel in the initial contrast-enhanced images compared to precontrast images.

On ultrafast MRI, four quantitative kinetic features were obtained using MATLAB (R2023b; MathWorks) by an MRI specialist (J.T.L.): TTE (s), maximum slope (percentage relative enhancement/second [%/s]), initial enhancement rate (a.u.), and U2 time (s) [21, 22]. A 3 × 3 mm circle region of interest (ROI) was placed on the most intensively enhancing region (hotspot) of segmented tumor (ROI_tumor_) based on a heatmap generated on the last ultrafast image. The same sized ROI was also placed on the descending aorta at the level of the main trunk of the pulmonary artery to evaluate the starting time of aortic enhancement as a reference (ROI_aorta_). Each ROI (ROI_tumor_ and ROI_aorta_) was applied to all other ultrafast MRI phases to generate time–signal intensity curves. The following kinetic parameters were derived from the time–signal intensity curves on ultrafast MRI. TTE was defined as the time interval between the timepoint at which the tumor begins to enhance minus the timepoint at which the aorta starts to enhance. It was formulated as TTE = (tumor enhancement phase − aorta enhancement phase) × repetition time. Maximum slope was defined as the slope of the tangent along the steepest part of the time–signal intensity curve. The tangent was calculated by connecting each time point, and the interval with the steepest tangent was decided for maximum slope from the time–signal intensity curve. Initial enhancement rate was defined as the ratio of change in signal intensity between the unenhanced phase and early phase. It was formulated as follows, initial enhancement rate = signal intensity of unenhanced phase/signal intensity of early phase. Among the 14 ultrafast images, U1 was the first phase in which the signal intensity of the hotspot ROI of the tumor was 10% higher than the average signal intensity of unenhanced images. U2 was the immediate next phase of U1 and the well-established tumor enhancement time [6]. U2 phase was reported as the optimal time to measure the maximum tumor size with little effect of background enhancement [23, 24]. Kinetic features were added to the radiomic features obtained from standard or ultrafast MRI for both readers.

**Fig. 3:**
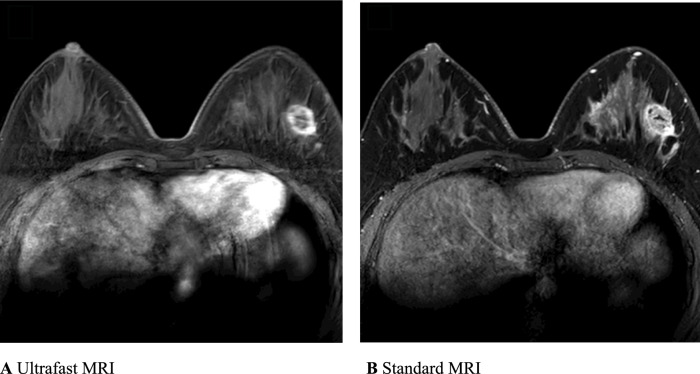
Ultrafast and standard MRI in a 30-year-old woman with triple-negative invasive ductal carcinoma. **A** Ultrafast MRI taken 16.8 s after contrast agent injection. **B** Standard MRI taken 58.8 s after contrast agent injection. Both images show an oval-shaped, irregular marginated, rim enhancing mass in the left outer breast. For three-dimensional tumor segmentation, the entire enhanced tumor margins were drawn from top to bottom of each tumor in axial views of U2 phase postcontrast ultrafast MRI and the initial phase postcontrast standard MRI. Among the 14 ultrafast images, U1 is the first phase in which the signal intensity of the hotspot region of interest of the tumor is 10% higher than the average signal intensity of unenhanced images. U2 is the immediate next phase of U1 and the well-established tumor enhancement time

Two readers (R1 [B.K.S.]: a dedicated breast radiologist with 24 years’ of experience in breast imaging, R2 [J.J.]: a radiology resident with 2 years’ of experience in breast imaging) performed background parenchymal enhancement assessments and tumor segmentation. Background parenchymal enhancement was classified into minimal, mild, moderate, or marked [25]. Readers were blinded to pathology reports and independently evaluated MRI after review of mammography and/or ultrasound images. They independently evaluated ultrafast and standard MRI images uploaded onto the Picture Archiving and Communication System in separate reading rooms. Standard and ultrafast image sets were uploaded and read with a one-month interval, respectively. For three-dimensional segmentation of tumors, ROIs along the entire enhancing tumor margin of cross-sectional area were drawn at axial views of standard and ultrafast images from top to bottom of each tumor by two radiologists. The initial phase contrast-enhanced images from standard MRI and U2 phase images from ultrafast MRI were chosen for tumor evaluation (Fig. 3). Three-dimensional segmentation of the tumor was performed using a semi-automated method with MRIcro software (version 1.40, https://www.nitrc.org/projects/mricro/). In cases of disagreement between the two radiologists on the classification of background parenchymal enhancement (*n* = 11) or the location of the segmented tumor (*n* = 2), a third radiologist (S.E.S.: a dedicated breast radiologist with 13 years’ of experience in breast imaging) reviewed the images and made the final decision.

Image normalization based on mean and standard deviation was performed prior to isotropic resampling to ensure consistency across all images. Isotropic resampling was performed before feature extraction to enhance the robustness of the features. A total of 1618 radiomic features of four groups were extracted from each cancer on both standard and ultrafast images: (a) first-order statistical features (*n* = 17), (b) shape and volume features (*n* = 7), (c) texture features using the gray-level co-occurrence matrix (GLCM) and gray-level run length matrix (*n* = 162), and (d) wavelet-transformed features (*n* = 1432) [14, 26]. Detailed feature evaluation is presented in Supplementary information. We extracted radiomic features in compliance with the image biomarker standardization initiative [27] using the Pyradiomics 3.1.0 library (https://www.radiomics.io/pyradiomics.html) in Python 3.8, with default setting. The radiomics quality score of this study was 25 (69%) out of 36 [14, 26] (Supplementary Table 1).

### Classification metrics and model construction

Medical records were reviewed to collect histological data. HR including estrogen and progesterone, and HER2 status and molecular subtypes were evaluated in surgical specimens from 174 participants who underwent surgery and core needle biopsy specimens from 51 participants who underwent neoadjuvant chemotherapy or palliative chemotherapy. The immunohistochemistry results for HR and HER2 were dichotomized as positive or negative. For HR status, greater than 2 of the Allred scoring system was considered positive [28]. HER2 overexpression was determined as 3 + immunohistochemistry staining or 2 + immunohistochemistry staining with HER2 gene amplification detected by silver-stained in situ hybridization. Molecular subtype was classified into three types according to St. Gallen classification criteria: luminal, HER2-overexpressed, or triple-negative [9].

The extracted radiomic and kinetic features were first subjected to z-score normalization. MRI feature selection was performed using the least absolute shrinkage and selection operator (LASSO) method, followed by fivefold cross-validation to determine the optimal lambda value of 0.05. Specifically, the dataset was divided into five equal-sized subsets; in each iteration of the cross-validation, four subsets were combined to form the training set, while the remaining subset was designated as the validation set. This cycle was repeated five times, and each subset was used only once as a validation set, comprehensively evaluating the performance of the model across the entire dataset as an average value. The LASSO process was repeated 25 times, and during each iteration, features that were selected 20 times were considered statistically significant as radiomic features. Additionally, an additional criterion was applied, where only features with a LASSO coefficient threshold of 0.6 and above were retained. Consequently, a final set of seven top features was identified. These features were used as predictors in the linear regression analysis [29, 30]. Moreover, the class weights in the linear regression models were adjusted based on the class frequencies in the input data. Specifically, each class's weight was set inversely proportional to its frequency, ensuring that samples from the minority class received higher weights compared to those from the majority class. The detailed classification metrics and model construction method are presented in Supplementary information.

### Statistical analysis

We tested the association between MRI radiomic features and histological factors and subtypes in standard and ultrafast MRI models. Heat maps were generated using Pearson correlation coefficients to visualize the linear associations between variables. The model performance of each reader was evaluated using an area under the receiver-operating characteristic curve (AUC), sensitivity, specificity, and accuracy using the Scikit-learn library in Python [31]. The AUC was calculated to assess the discriminatory power of the model, and 95% confidence intervals (CI) were calculated to estimate the uncertainty and precision of the AUC estimate. The DeLong test was used to determine any differences between the two models based on the receiver-operating characteristic curves.

**Fig. 4:**
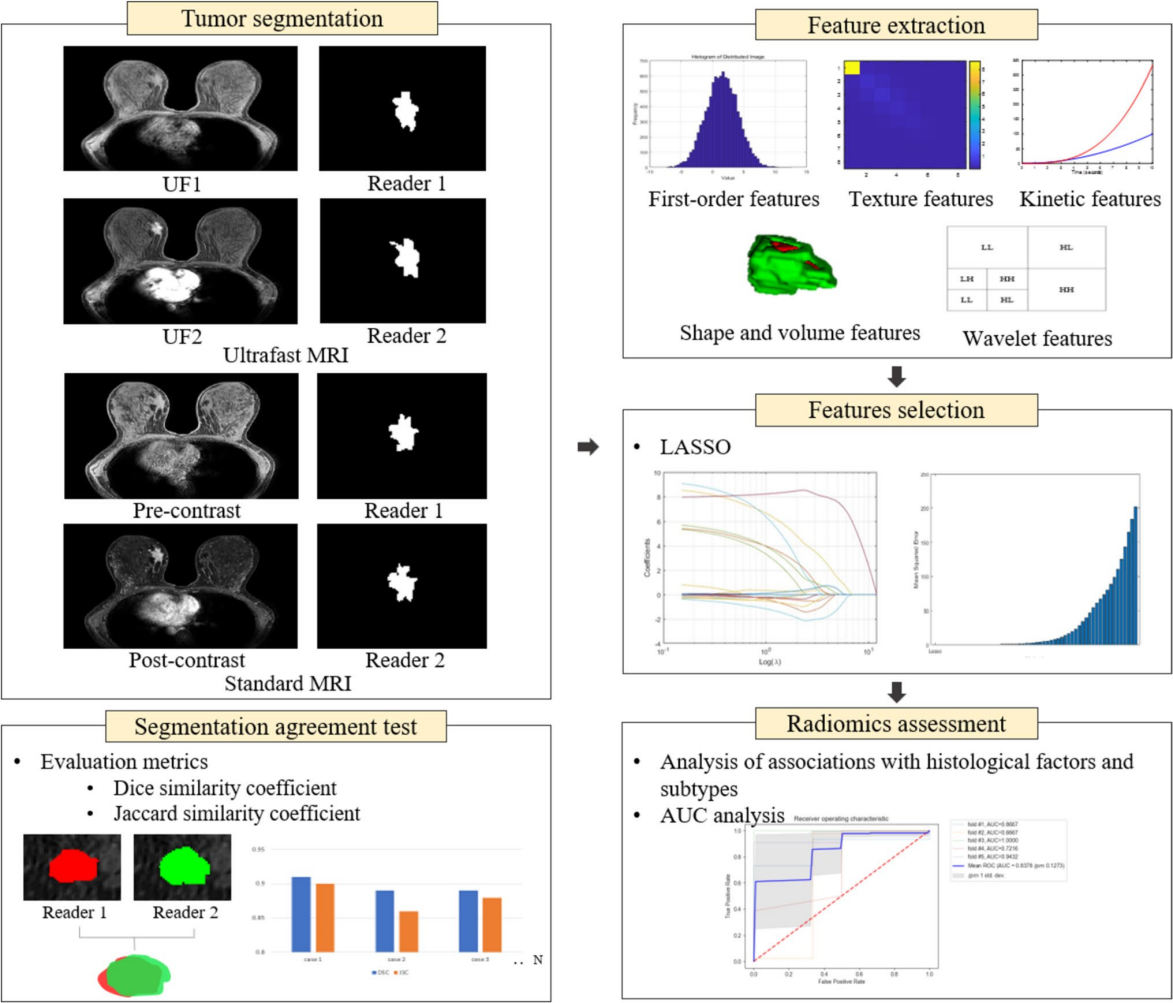
Illustration of the radiomics workflow. Three-dimensional tumor segmentation was performed on ultrafast MRI and standard MRI by two readers (Reader 1: a dedicated breast radiologist, Reader 2: a radiology resident), independently. The tumor segmentation agreement between two readers was evaluated using Dice and Jaccard similarity coefficients. After segmentation, 1618 radiomic features (first-order statistical features, shape and volume features, texture features, and wavelet-transformed features) were extracted. Kinetic features obtained from ultrafast MRI and standard MRI were added to the radiomic features. MRI feature selection was performed using the LASSO method. We tested the associations between MRI radiomic features and histological factors and subtypes in two models of ultrafast and standard MRI. The model performance of each reader was evaluated using the AUC analysis. *LASSO* least absolute shrinkage and selector operator, *AUC* the area under the receiver-operating characteristic curve

We also evaluated the agreement between the segmentations of R1 and R2 using the Dice similarity coefficient and the Jaccard similarity coefficient (MATLAB R2023b) [32–34]. When segmenting a ROI in an image, it is essential to assess the consistency between multiple readers given the potential subjectivity of the segmentation. The Dice similarity coefficient quantifies the overlap between two sets, with a value of 1 indicating perfect overlap and 0 indicating no overlap. Similarly, the Jaccard similarity coefficient measures the ratio of the intersection to the union of two sets, where a score of 1 denotes complete agreement and 0 means no overlap. Statistical significance was determined using a *P* value threshold of less than 0.05. The overall research flow of the study is presented in Fig. 4.

## Results

### Study participants

Table 2 demonstrates 225 participants’ characteristics with 233 invasive breast cancers. They were all women (mean age, 54 years ± 12 [SD]). Mean tumor size on MRI was 25 ± 16 mm. Histological types of cancers confirmed as invasive ductal carcinoma (*n* = 207), invasive lobular carcinoma (*n* = 12), mucinous carcinoma (*n* = 4), and others (*n*$$ = 10$$).

**Table 2:** Study participant characteristics

Characteristics	Value
Age (years)	54 ± 11 (27–86)
Tumor size (mm)	25 ± 16 (6–115)
Background parenchymal enhancement on MRI	
Minimal	102 (44%)
Mild	57 (25%)
Moderate	37 (16%)
Marked	37 (16%)
Lesion type on MRI	
Mass	212 (91%)
Nonmass enhancement	21 (9%)
Histological type	
Invasive ductal carcinoma	207 (89%)
Invasive lobular carcinoma	12 (5%)
Mucinous carcinoma	4 (2%)
Others	10 (3%)
Molecular subtype	
Luminal (HR $+$)	186 (80%)
HER2-overexpressed (HR $-$, HER2 $+$)	17 (7%)
Triple-negative (HR $-,$ HER2 $-$)	30 (13%)
Immunohistochemistry	
HR positivity	186 (80%)
HER2 positivity	52 (22%)

225 participants with 233 invasive breast cancers were included in this study. All participants (*n* = 225) were women. Unless otherwise indicated, data are number of cancers with percentage in parentheses. Age and tumor size are presented with mean value ± standard deviation, and data in parentheses are range. Tumor size was measured on U2 phase of ultrafast MRI. *HR* hormone receptor, *HER2* human epidermal growth factor receptor 2

### Tumor segmentation agreement between two readers

**Fig. 5:**
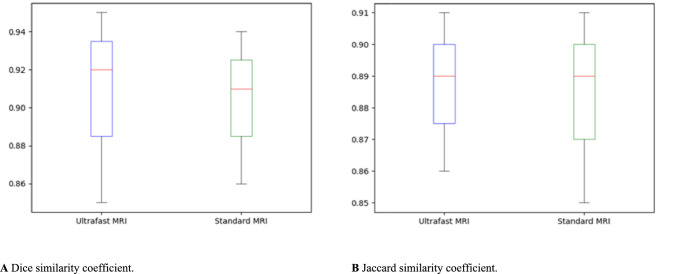
Tumor segmentation agreement between two readers

The tumor segmentation agreement between two readers is shown in Fig. 5. On ultrafast MRI, Dice similarity coefficient was 0.91 ± 0.06 and Jaccard similarity coefficient was 0.89 ± 0.04. On standard MRI, Dice similarity coefficient was measured as 0.92 ± 0.06 and Jaccard similarity coefficient as 0.89 ± 0.05.

### Tumor classification performances according to histological factors and subtypes

Table 3 shows the classification performance according to histological factors using radiomic signatures. In classifying HER2 status, ultrafast MRI outperformed standard MRI for both readers (AUCs [95% CI] of ultrafast MRI vs standard MRI; 0.87 [0.83–0.91] vs 0.77 [0.64–0.90] for R1 and 0.88 [0.83–0.91] vs 0.77 [0.69–0.84] for R2) (all *P* < 0.05). In classifying HR status, ultrafast MRI performance was higher than standard MRI (AUCs [95% CI] of ultrafast MRI vs standard MRI; 0.80 [0.74–0.86] vs 0.77 [0.70–0.84] for R1 and 0.77 [0.70–0.84] vs 0.73 [0.65–0.80] for R2); however, this was not statistically significant (*P* = 0.07 for R1 and *P* = 0.05 for R2). Supplementary Table 2 demonstrates the radiomic signatures to classify each histological factor. Among 14 radiomic signatures for classifying HR or HER2 status on ultrafast MRI, there were ten first-order, three ultrafast kinetic, and one shape and volume features for both readers. On standard MRI, radiomic signatures were seven GLCM and seven first-order features for R1 and eight GLCM and six first-order features for R2.

**Table 3:** Classification performance of histological factors

Reader	MRI modality	Histological factors	Accuracy (%)	Sensitivity (%)	Specificity (%)	AUC	*P* value*
R1	Ultrafast	HR	0.82 (0.76–0.88)	0.85 (0.80–0.89)	0.55 (0.50–0.60)	0.80 (0.74–0.86)	0.07
		HER2	0.83 (0.77–0.87)	0.83 (0.78–0.88)	0.70 (0.64–0.76)	0.87 (0.83–0.91)	0.02
	Standard	HR	0.81 (0.76–0.86)	0.85 (0.80–0.90)	0.68 (0.61–0.75)	0.77 (0.70–0.84)	
		HER2	0.75 (0.68–0.82)	0.73 (0.67–0.78)	0.70 (0.58–0.77)	0.77 (0.64–0.90)	
R2	Ultrafast	HR	0.81 (0.74–0.88)	0.81 (0.75–0.85)	0.68 (0.61–0.75)	0.77 (0.70–0.84)	0.05
		HER2	0.83 (0.77–0.87)	0.83 (0.78–0.88)	0.71 (0.65–0.77)	0.88 (0.83–0.91)	0.03
	Standard	HR	0.78 (0.72–0.84)	0.82 (0.77–0.87)	0.61 (0.52–0.70)	0.73 (0.65–0.80)	
		HER2	0.74 (0.62–0.85)	0.71 (0.61–0.89)	0.72 (0.63–0.80)	0.77 (0.69–0.84)	

Data are given as medians and 95% confidence intervals in parentheses. *P values are provided by DeLong’s test to compare AUC values between ultrafast MRI and standard MRI for each reader. *HR* hormone receptor, *HER2* human epidermal growth factor receptor 2. *AUC* area under the receiver-operating characteristic curve

Table 4 shows the classification performance of molecular subtypes using radiomic signatures. The performance of ultrafast MRI surpassed that of standard MRI in classifying all subtypes (luminal, HER2-overexpressed, and triple-negative) for both readers. Specifically, when classifying the luminal subtype, this difference was statistically significant (AUCs [95% CI] of ultrafast MRI vs standard MRI; 0.88 [0.82–0.94] vs 0.83 [0.72–0.88] for R1 and 0.82 [0.75–0.88] vs 0.81 [0.75–0.87] for R2) (all *P* = 0.04). In classifying the HER2-overexpressed subtype, the performance difference was statistically significant for R2 (AUCs [95% CI] of ultrafast MRI vs standard MRI; 0.79 [0.74–0.84] vs 0.77 [0.67–0.86] for R1 and 0.81 [0.76–0.86] vs 0.76 [0.65–0.87] for R2) (*P* = 0.07 for R1 and *P* = 0.04 for R2). Additionally, in classifying the triple-negative subtype, ultrafast MRI outperformed standard MRI (AUCs [95% CI] of ultrafast MRI vs standard MRI; 0.78 [0.69–0.84] vs 0.71 [0.65–0.77] for R1 and 0.76 [0.69–0.83] vs 0.73 [0.67–0.78] for R2) (*P* = 0.04 for R1 and *P* = 0.05 for R2). Supplementary Table 3 shows the radiomic signatures for classifying subtypes. Among 21 radiomic features on ultrafast MRI, there were nine first-order, six GLCM, three shape and volume, two ultrafast kinetic features, and one gray-level run length matrix features for R1, and nine GLCM, seven first-order, four shape and volume, and one ultrafast kinetic feature for R2. On standard MRI, radiomic signatures were 14 GLCM, six first-order, one shape and volume features for R1 and 16 texture, and five first-order features for R2. Among ultrafast MRI kinetic features, TTE was most frequently chosen radiomic signature when classifying histological factors and molecular subtypes for both readers. No standard MRI kinetic features were selected as radiomic signatures.

**Table 4:** Classification performance of molecular subtypes

Reader	MRI modality	Subtype	Accuracy (%)	Sensitivity (%)	Specificity (%)	AUC	*P* value*
R1	Ultrafast	Luminal	0.85 (0.80–0.90)	0.87 (0.81–0.93)	0.69 (0.64–0.76)	0.88 (0.82–0.94)	0.04
		HER2-overexpressed	0.87 (0.82–0.91)	0.89 (0.84–0.92)	0.60 (0.54–0.66)	0.79 (0.74–0.84)	0.07
		Triple-negative	0.86 (0.81–0.90)	0.88 (0.83–0.92)	0.70 (0.64–0.75)	0.78 (0.69–0.84)	0.04
	Standard	Luminal	0.81 (0.74–0.88)	0.82 (0.73–0.92)	0.69 (0.62–0.76)	0.83 (0.72–0.88)	
		HER2-overexpressed	0.84 (0.75–0.92)	0.87 (0.77–0.91)	0.69 (0.60–0.77)	0.77 (0.67–0.86)	
		Triple-negative	0.81 (0.73–0.84)	0.78 (0.75–0.85)	0.57 (0.40–0.53)	0.71 (0.65–0.77)	
R2	Ultrafast	Luminal	0.83 (0.77–0.89)	0.85 (0.80–0.88)	0.66 (0.62–0.72)	0.82 (0.75–0.88)	0.04
		HER2-overexpressed	0.82 (0.76–0.86)	0.82 (0.76–0.86)	0.73 (0.67–0.79)	0.81 (0.76–0.86)	0.04
		Triple-negative	0.83 (0.78–0.87)	0.84 (0.79–0.88)	0.70 (0.64–0.76)	0.76 (0.69–0.83)	0.05
	Standard	Luminal	0.81 (0.74–0.88)	0.79 (0.70–0.85)	0.65 (0.56–0.74)	0.81 (0.75–0.87)	
		HER2-overexpressed	0.82 (0.76–0.88)	0.85 (0.76–0.91)	0.72 (0.65–0.79)	0.76 (0.65–0.87)	
		Triple-negative	0.78 (0.66–0.89)	0.80 (0.71–0.89)	0.77 (0.63–0.89)	0.73 (0.67–0.78)	

Data are given as medians and 95% confidence intervals in parentheses. *P values are provided by DeLong’s test to compare AUC values between ultrafast MRI and standard MRI for each reader. *HER2* human epidermal growth factor receptor 2, *AUC* area under the receiver-operating characteristic curve

**Fig. 6:**
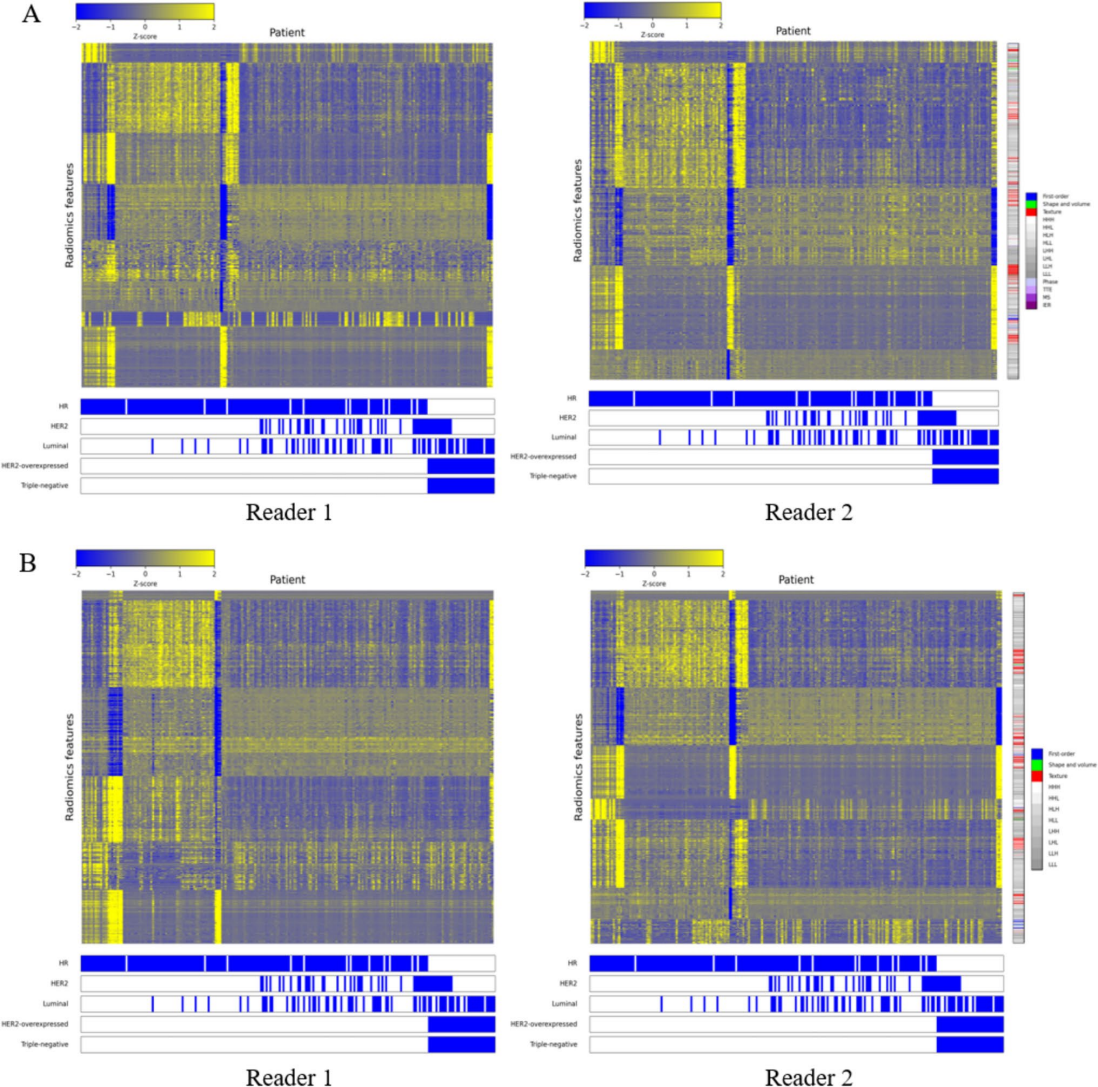
Radiomics heat map. Heat maps show associations between radiomic features from ultrafast MRI (**A**) and standard MRI (**B**) in Reader 1 and Reader 2, respectively, and histological factors and molecular subtypes. Maps illustrate the variation in signal intensity across multiple features, with each row representing a specific feature. A color intensity scale from low (blue) to high (yellow) is provided. Histograms below each heat map detail the distribution of specific histological markers, including HR and HER2 status, and luminal, HER2-overexpressed, and triple-negative subtypes. These markers were dichotomized into binary categories, denoted as 0 or 1

Heat maps in Fig. 6 show that the regions corresponding to first-order and texture features predominantly showed high intensities, while the regions related to wavelet features showed somewhat lower intensities. Additionally, ultrafast MRI displayed more pronounced intensities than standard MRI, allowing for clearer distinctions.

## Discussion

Our prospective radiomics study demonstrates that ultrafast MRI exhibits superior classification performance for breast cancer HER2 status and molecular subtype compared to standard MRI for radiologists of different experience levels (a dedicated breast radiologist and a radiology resident). Notably, ultrafast MRI outperformed standard MRI in predicting all subtypes including luminal, HER2-overexpressed, and triple-negative. Additionally, both readers showed high agreement for tumor segmentation in both MRI modalities, as indicated by Dice and Jaccard similarity coefficients. Consequently, our study underscores the potential clinical utility of ultrafast MRI radiomics in breast cancer classification, irrespective of readers' experience levels.

Ultrafast MRI is taken with high temporal resolution and completed before the initial postcontrast images of a standard dynamic MRI begin. Previous studies have validated its use in accurate evaluation of tumor size with good lesion conspicuity [5, 6]. Furthermore, ultrafast MRI produces reproducible quantitative kinetic features [21, 35]. In this study, TTE was most frequently selected as a radiomics signature among four kinetic features to classify HR and HER2 status and luminal and triple-negative subtypes. TTE indicates the time interval between when the tumor begins to enhance and when the aorta begins to enhance. Previous studies have shown that shorter TTE is valuable to differentiate malignant from benign breast lesions and correlates with tumor aggressiveness, high grade, HR negativity, HER2 positivity, and triple-negative subtype [21, 36–38]. Therefore, our results are consistent with previous studies.

On standard MRI, the association between HER2 status and radiomics, as well as the relationship between molecular subtypes and radiomics, has been reported [39–42]. This year, Ramtohul et al. [39] demonstrated high performance (AUC [95% CI] = 0.80 [0.71–0.89]) in differentiating HER2-zero cancers vs HER2-low and positive cancers. In recent studies on the prediction of triple-negative cancers, reported AUC values ranged from 0.73 to 0.88 in radiomics models using standard MRI [41, 42]. Apart from luminal subtypes, which tend to respond well to endocrine therapy, HER2 overexpression causes uncontrolled cell growth and is associated with higher recurrence and mortality in the absence of targeted therapy [43], while triple-negative breast cancers can result in the worst prognosis as they can only be treated with chemotherapy and radiotherapy. Therefore, our findings that ultrafast MRI outperforms standard MRI in classifying HER2 status and all molecular subtypes raise expectations for the clinical applicability of ultrafast MRI radiomics in breast cancer.

In radiomics, agreement on tumor segmentation among various readers is important to increase reproducibility and generalizability. Granzier et al. [44] evaluated segmentation variability among four readers with different breast imaging experiences (a dedicated breast radiologist, a radiology resident, a medical student, and a PhD. candidate) and observed a Dice similarity coefficient of 0.81, indicating a good spatial overlap regardless of different expertise. In our study, the Dice similarity coefficients of ultrafast MRI (0.91) and standard MRI (0.92) were higher than that of the previous study [44]. Due to the high agreement on tumor segmentation between the two readers in our study, the selected radiomic signatures for both readers were similar. For instance, when classifying histological factors, the majority of radiomic signatures consisted of first-order features on ultrafast MRI and GLCM features on standard MRI for both readers. High segmentation agreement between various MRI readers increases the robustness of radiomics and improves the reproducibility and generalizability of artificial intelligence-assisted interpretation.

Our prospective study contributes to the field of breast cancer radiomics and ultrafast MRI research. First, there are a few reports applying radiomics to ultrafast MRI and our radiomics results demonstrate that ultrafast MRI can be an alternative to the standard MRI in breast oncology imaging. Second, we evaluated breast cancer classification performance for all molecular subtypes and demonstrated superior performance compared to standard MRI. Third, we designed a high-quality radiomics study to obtain robust radiomics results. Our radiomics quality score was high, 69% (25/36), due to prospective study design, segmentation by different readers, and open-source analyzing software [14, 26]. A previous study reported the mean radiomics quality score of oncology imaging of 26% (9/36) [14].

Our study has some limitations. First, we did not evaluate the impact of individual MRI characteristics, such as tumor size or morphological features, on classification performance. For example, the tumor sizes in this study ranged from 6 to 115 mm, showing considerable variability. Predicting performance evaluation according to the size subcategory would be necessary for clinical implementation. Second, we did not include cancers smaller than 6 mm in size or inflammatory cancers. Inflammatory breast cancer involves the skin and dermal lymphatics, making it challenging to delineate tumor margins. A previous study demonstrates that “easy tumors,” defined as homogenous, round tumors with relatively sharp margins have better agreement than “challenging tumors” that do not meet these criteria [44]. To improve the clinical utility of ultrafast MRI radiomics for breast cancer classification, further research on the impact of various lesion characteristics should be conducted using a larger population, including tumors of various morphologies and sizes.

In conclusion, ultrafast MRI-based radiomics can serve as a noninvasive tool to classify breast cancer according to histological factors and subtypes as compared with standard MRI. Ultrafast MRI exhibited superior performance to standard MRI in classifying HER2 status, as well as luminal, HER2-overexpressed, and triple-negative molecular subtypes. These results are consistent regardless of the experience of the radiologists. Our study demonstrates the promise of radiomics approaches with ultrafast MRI that have advantages in terms of scan time and lesion conspicuity, which are problems with standard MRI.

## Supplementary Information

Below is the link to the electronic supplementary material.

Supplementary file1 (DOCX 52 kb)

## Data Availability

The datasets used in the current study are available from the corresponding author on reasonable request.
