# Supplementary material for: Superior performance in classification of breast cancer molecular subtype and histological factors by radiomics based on ultrafast MRI over standard MRI: evidence from a prospective study
Source: Radiol Med. 2025 Jan 25;130(3):368–80. doi: 10.1007/s11547-025-01956-6 (PMC11903601; doi:10.1007/s11547-025-01956-6)
Supplement: Supplementary file 1 — Supplementary file1 (DOCX 52 kb) [file 11547_2025_1956_MOESM1_ESM.docx]

**Supplementary Information**

**Sample size determination**

Few studies have evaluated the performance of ultrafast MRI radiomics, and sample size was determined based on a pilot study comparing performance between ultrafast MRI radiomics and standard MRI radiomics [1]. A sample of 75 from the positive group and 150 from the negative group achieved 80% power to detect a difference of 0.04 between a diagnostic test with an area under the receiver-operating characteristic curve (AUC) of 0.67 and another diagnostic test with an AUC of 0.71 using a two-sided z-test at a significance level of 0.05. The correlation between the two diagnostic tests was assumed to be 0.95 for the positive group and 0.95 for the negative group. The dropout rate was assumed to be 0.15 for subjects that are expected to be lost at random during the study.

**Radiomics feature evaluation**

Radiomic features were categorized into four groups: (1) first-order statistical features (*n* $=$ 17), (2) shape and volume features (*n* $=$ 7), (3) texture features using the gray-level co-occurrence matrix (GLCM) and gray-level run length matrix (GLRLM) (*n* $=$ 162), and (4) wavelet-transformed features (*n*$=$ 1432).

(1) First- order statistical features were computed directly from the intensity values of the histogram within ROI and provided insights into the distribution and statistical properties of voxel intensities.

(2) Shape and volume features were calculated to capture information about the shape and size of the ROIs.

(3) Texture features captured the texture characteristics and structural patterns of the volume within the ROI, and GLCM and GLRLM were used to calculate the spatial dependencies, and irregularities of the image voxels.

(4) Wavelet features were extracted from the transformed wavelet space by generating wavelet decomposition images through eight high- and low-pass filters, LLH, LHL, LHH, LLL, HLL, HLH, HHL and HHH, in each ROI, where “L” represents “low-pass filter” and “H” represents “high-pass filter.” We then applied the first-order features and texture features to the wavelet-transformed image.

**Supplementary Table 1** The radiomics quality score of this study

| Domain |  |  | Score | RQS Criteria | Our Score |
| --- | --- | --- | --- | --- | --- |
| 1 | Image protocol quality | Well-documented image protocols (for example, contrast, slice thickness, energy, etc.) and/or usage of public image protocols allow reproducibility/replicability | + 1 (if protocols are well-documented)  + 1 (if public protocol is used) | 1 | 2 |
|  | Multiple segmentations | Segmentation by different physicians/algorithms/software, perturbing segmentations by (random) noise, segmentation at different breathing cycles. Analyse feature robustness to segmentation variabilities | + 1 | 2 | 1 |
|  | Phantom study on all scanners | Detect inter-scanner differences and vendor-dependent features. Analyse feature robustness to these sources of variability | + 1 | 3 | 0 |
|  | Imaging at multiple time points | Collect images of individuals at additional time points. Analyse feature robustness to temporal variabilities (for example, organ movement, organ expansion/ shrinkage) | + 1 | 4 | 0 |
| 2 | Feature reduction or adjustment for multiple testing | Decreases the risk of overfitting. Overfitting is inevitable if the number of features exceeds the number of samples. Consider feature robustness when selecting features | - 3 (if neither measure is implemented) + 3 (if either measure is implemented) | 5 | 3 |
|  | Validation | The validation is performed without retraining and without adaptation of the cut-off value, provides crucial information with regards to credible clinical performance | - 5 (if validation is missing)  + 2 (if validation is based on a dataset from the same institute)  + 3 (if validation is based on a dataset from another institute)  + 4 (if validation is based on two datasets from two distinct institutes)  + 4 (if the study validates a previously published signature)  + 5 (if validation is based on three or more datasets from distinct institutes) | 12 | 2 |
| 3 | Multivariable analysis with non-radiomics features | (for example, EGFR mutation) - is expected to provide a more holistic model. Permits correlating/inferencing between radiomics and non radiomics features | +1 | 6 | 0 |
|  | Detect and discuss biological correlates | Demonstration of phenotypic differences (possibly associated with underlying gene–protein expression patterns) deepens understanding of radiomics and biology | +1 | 7 | 1 |
|  | Comparison to ‘gold standard’ | Assess the extent to which the model agrees with/is superior to the current ‘gold standard’ method (for example, TNM-staging for survival prediction). This comparison shows the added value of radiomics | +2 | 13 | 2 |
|  | Potential clinical utility | Report on the current and potential application of the model in a clinical setting (for example, decision curve analysis). | +2 | 14 | 0 |
| 4 | Cut-off analyses | Determine risk groups by either the median, a previously published cut-off or report a continuous risk variable. Reduces the risk of reporting overly optimistic results | +1 | 8 | 0 |
|  | Discrimination statistics | Report discrimination statistics (for example, C‑statistic, ROC curve, AUC) and their statistical significance (for example, p‑values, confidence intervals). One can also apply resampling method (for example, bootstrapping, cross-validation) | + 1 (if a discrimination statistic and its statistical significance are reported) + 1 (if a resampling method technique is also applied) | 9 | 2 |
|  | Calibration statistics | Report calibration statistics (for example, Calibration-in‑the-large/slope, calibration plots) and their statistical significance (for example, *P*‑values, confidence intervals). One can also apply resampling method (for example, bootstrapping, cross-validation) | + 1 (if a calibration statistic and its statistical significance are reported) + 1 (if a resampling method technique is also applied) | 10 | 2 |
| 5 | Prospective study registered in a trial database | Provides the highest level of evidence supporting the clinical validity and usefulness of the radiomics biomarker | + 7 (for prospective validation of a radiomics signature in an appropriate trial) | 11 | 7 |
|  | Cost-effectiveness analysis | Report on the cost-effectiveness of the clinical application (for example, QALYs generated) | +1 | 15 | 0 |
| 6 | Open science and data | Make code and data publicly available. Open science facilitates knowledge transfer and reproducibility of the study | + 1 (if scans are open source) + 1 (if region of interest segmentations are open source) + 1 (if code is open source) + 1 (if radiomics features are calculated on a set of representative ROIs and the calculated features and representative ROIs are open source) | 16 | 3 |

The radiomics quality score is a system metrics for evaluating quality of radiomics proposed by Lambin et al. [2]. It consists of 16 items, with a total score ranging from $-8 to+$36 points.

**Supplementary Table 2** Radiomic signatures to classify histological factors

| Reader | MRI Modality | HR | HER2 |
| --- | --- | --- | --- |
| R1 | Ultrafast | Shape/Volume_Compactness | First-order_Median |
|  |  | First-order_Sum | First-order_Kurtosis |
|  |  | Ultrafast_TTE | Ultrafast_TTE |
|  |  | First-order_Range | First-order_Counts |
|  |  | First-order_Median | First-order_Variance |
|  |  | First-order_Kurtosis | Ultrafast_MS |
|  |  | First-order_Minimum | First-order_Sum |
|  | Standard | First-order_Range | First-order_Maximum |
|  |  | GLCM_Harralick correlation | GLCM_Contrast_std |
|  |  | First-order_Kurtosis | GLCM_Cluster prominence |
|  |  | GLCM_Harralick correlation_std | First-order_Kurtosis |
|  |  | First-order_Skewness | GLCM_Difference entropy_std |
|  |  | GLCM_Sum entropy | GLCM_Autocorrelation |
|  |  | First-order_Maximum | First-order_Median |
| R2 | Ultrafast | First-order_Sum | First-order_Median |
|  |  | Shape/Volume_Compactness | First-order_Kurtosis |
|  |  | First-order_Mean | Ultrafast_TTE |
|  |  | Ultrafast_U2 phase | First-order_Counts |
|  |  | First-order_Min | First-order_Variance |
|  |  | First-order_RMS | First-order_Std |
|  |  | Ultrafast_TTE | First-order_Sum |
|  | Standard | First-order_Kurtosis | GLCM_Dissimilarity |
|  |  | GLCM_Energy | GLCM_Cluster prominence |
|  |  | GLCM_ Dissimilarity | First-order_Minimum |
|  |  | GLCM_Difference variance average | First-order_Kurtosis |
|  |  | GLCM_ Sum of sqaures | GLCM_Difference average |
|  |  | GLCM_Harralick correlation | First-order_Sum |
|  |  | First-order_Min | First-order_Range |

The radiomic feature name is described by group_discriptor_statistic. *GLCM* gray-level co-occurrence matrix, *Std* standard deviation, *RMS* root mean square, *MS* maximum slope, *TTE* time to enhancement.

**Supplementary Table 3** Radiomic signatures to classify molecular subtypes

| Reader | MRI Modality | Luminal | HER2-overexpressed | Triple-negative |
| --- | --- | --- | --- | --- |
| R1 | Ultrafast | First-order_Range | GLRLM_LRHGE_std | First-order_Mean |
|  |  | First-order_Mean | First-order_Mad | Ultrafast_TTE |
|  |  | First-order_Std | GLCM_Inverse difference normalized_std | First-order_Median |
|  |  | GLCM_Sum variance | GLCM_Homogeneity_std | First-order_Std |
|  |  | Shape/Volume_Compactness | GLCM_Maximum probability | First-order_Uniformity |
|  |  | Ultrafast_TTE | GLCM_Cluster prominence_std | Shape/Volume_Surface |
|  |  | First-order_Uniformity | GLCM_Correlation | Shape/Volume_Compactness |
|  | Standard | GLCM_Energy | First-order_Kurtosis | GLCM_Sum of sqaures_std |
|  |  | First-order_Minimum | GLCM_Energy | GLCM_Sum of sqaures |
|  |  | GLCM_Correlation | GLCM_Difference variance_std | GLCM_Dissimilarity |
|  |  | First-order_Uniformity | GLCM_Cluster tendenc | Shape/Volume_Compactness |
|  |  | GLCM_Harralick correlation | GLCM_Difference entropy | GLCM_Cluster shade_std |
|  |  | First-order_Maximum | GLCM_Homogeneity | GLCM_Energy |
|  |  | First-order_Sum | GLCM_Difference average_std | First-order_Range |
| R2 | Ultrafast | First-order_Std | Shape/Volume_surface | First-order_Mean |
|  |  | First-order_Energy | GLCM_Maximum probability | GLCM_Correlation |
|  |  | First-order_Mean | GLCM_Maximum probability_std | Ultrafast_U2 phase |
|  |  | First-order_Sum | GLCM_Correlation | First-order_Std |
|  |  | Shape/Volume_Compactness | GLCM_Dissimilarity | Shape/Volume_Compactness |
|  |  | First-order_Range | GLCM_Maximum probability | GLCM_Difference entropy |
|  |  | GLCM_Contrast | GLCM_Cluster prominence_std | Shape/Volume_Surface |
|  | Standard | GLCM_Homogeneity_std | First-order_Kurtosis | GLCM_Sum of sqaures_std |
|  |  | First-order_Uniformity | GLCM_Energy | GLCM_Sum of sqaures |
|  |  | GLCM_Energy | GLCM_Cluster tendenc_std | GLCM_Entropy_std |
|  |  | First-order_Min | GLCM_Difference average | GLCM_Homogeneity_std |
|  |  | GLCM_Sum entropy_std | GLCM_Difference entropy | GLCM_Entropy_std |
|  |  | First-order_Skewness | GLCM_Difference average_std | GLCM_Sum variance |
|  |  | GLCM_Harralick correlation | GLCM_Difference variance | First-order_Skewness |

The radiomic feature name is described by group_discriptor_statistic. *GLCM* gray-level co-occurrence matrix, *GLRLM* gray level run length matrix, *Std* standard deviation, *TTE* time to enhancement, *LRHGE* long run high gray-level emphasis.

**Supplementary Table 4** Contingency table results for each fold of 5-fold cross validation

| Reader 1, Ultrafast | HR | | | |
| --- | --- | --- | --- | --- |
|  | TP | TN | FP | FN |
| Fold 1 | 158 | 28 | 19 | 28 |
| Fold 2 | 162 | 30 | 17 | 24 |
| Fold 3 | 160 | 32 | 15 | 26 |
| Fold 4 | 155 | 40 | 7 | 31 |
| Fold 5 | 159 | 30 | 17 | 27 |
| Reader 1, Ultrafast | HER2 | | | |
|  | TP | TN | FP | FN |
| Fold 1 | 35 | 157 | 24 | 17 |
| Fold 2 | 39 | 148 | 33 | 13 |
| Fold 3 | 41 | 152 | 29 | 11 |
| Fold 4 | 40 | 160 | 21 | 12 |
| Fold 5 | 38 | 166 | 15 | 14 |
| Reader 1, Ultrafast | Luminal | | | |
|  | TP | TN | FP | FN |
| Fold 1 | 162 | 32 | 15 | 24 |
| Fold 2 | 166 | 35 | 12 | 20 |
| Fold 3 | 161 | 30 | 17 | 25 |
| Fold 4 | 160 | 33 | 14 | 26 |
| Fold 5 | 165 | 34 | 13 | 21 |
| Reader 1, Ultrafast | HER2-overexpressed | | | |
|  | TP | TN | FP | FN |
| Fold 1 | 13 | 191 | 25 | 4 |
| Fold 2 | 11 | 188 | 28 | 6 |
| Fold 3 | 11 | 198 | 18 | 6 |
| Fold 4 | 12 | 179 | 37 | 5 |
| Fold 5 | 14 | 187 | 29 | 3 |
| Reader 1, Ultrafast | Triple-negative | | | |
|  | TP | TN | FP | FN |
| Fold 1 | 26 | 160 | 43 | 4 |
| Fold 2 | 24 | 155 | 48 | 6 |
| Fold 3 | 25 | 184 | 19 | 5 |
| Fold 4 | 23 | 179 | 24 | 7 |
| Fold 5 | 26 | 174 | 29 | 4 |
| Reader 1, Standard | HR |  |  |  |
|  | TP | TN | FP | FN |
| Fold 1 | 150 | 25 | 22 | 36 |
| Fold 2 | 159 | 26 | 21 | 27 |
| Fold 3 | 163 | 28 | 19 | 23 |
| Fold 4 | 155 | 30 | 17 | 31 |
| Fold 5 | 149 | 29 | 18 | 37 |
| Reader 1, Standard | HER2 | | | |
|  | TP | TN | FP | FN |
| Fold 1 | 30 | 141 | 40 | 22 |
| Fold 2 | 35 | 150 | 31 | 17 |
| Fold 3 | 38 | 136 | 45 | 14 |
| Fold 4 | 36 | 138 | 43 | 16 |
| Fold 5 | 39 | 129 | 52 | 13 |
| Reader 1, Standard | Luminal | | | |
|  | TP | TN | FP | FN |
| Fold 1 | 159 | 30 | 17 | 27 |
| Fold 2 | 157 | 26 | 21 | 29 |
| Fold 3 | 150 | 29 | 18 | 36 |
| Fold 4 | 161 | 31 | 16 | 25 |
| Fold 5 | 158 | 34 | 13 | 28 |
| Reader 1, Standard | HER2-overexpressed | | | |
|  | TP | TN | FP | FN |
| Fold 1 | 10 | 173 | 43 | 7 |
| Fold 2 | 14 | 189 | 27 | 3 |
| Fold 3 | 11 | 188 | 28 | 6 |
| Fold 4 | 11 | 180 | 36 | 6 |
| Fold 5 | 12 | 183 | 33 | 5 |
| Reader 1, Standard | Triple-negative | | | |
|  | TP | TN | FP | FN |
| Fold 1 | 25 | 128 | 75 | 5 |
| Fold 2 | 24 | 144 | 59 | 6 |
| Fold 3 | 21 | 189 | 14 | 9 |
| Fold 4 | 22 | 157 | 46 | 8 |
| Fold 5 | 19 | 180 | 23 | 11 |
| Reader 2, Ultrafast | HR |  |  |  |
|  | TP | TN | FP | FN |
| Fold 1 | 149 | 20 | 27 | 37 |
| Fold 2 | 157 | 26 | 21 | 29 |
| Fold 3 | 162 | 36 | 11 | 24 |
| Fold 4 | 159 | 32 | 15 | 27 |
| Fold 5 | 152 | 24 | 23 | 34 |
| Reader 2, Ultrafast | HER2 | | | |
|  | TP | TN | FP | FN |
| Fold 1 | 33 | 168 | 13 | 19 |
| Fold 2 | 38 | 172 | 9 | 14 |
| Fold 3 | 28 | 154 | 27 | 24 |
| Fold 4 | 34 | 176 | 5 | 18 |
| Fold 5 | 25 | 138 | 43 | 27 |
| Reader 2, Ultrafast | Luminal | | | |
|  | TP | TN | FP | FN |
| Fold 1 | 160 | 29 | 18 | 26 |
| Fold 2 | 162 | 30 | 17 | 24 |
| Fold 3 | 161 | 31 | 16 | 25 |
| Fold 4 | 159 | 32 | 15 | 27 |
| Fold 5 | 158 | 35 | 12 | 28 |
| Reader 2, Ultrafast | HER2-overexpressed | | | |
|  | TP | TN | FP | FN |
| Fold 1 | 12 | 174 | 42 | 5 |
| Fold 2 | 9 | 185 | 31 | 8 |
| Fold 3 | 10 | 184 | 32 | 7 |
| Fold 4 | 11 | 179 | 37 | 6 |
| Fold 5 | 13 | 188 | 28 | 4 |
| Reader 2, Ultrafast | Triple-negative | | | |
|  | TP | TN | FP | FN |
| Fold 1 | 23 | 156 | 47 | 7 |
| Fold 2 | 25 | 148 | 55 | 5 |
| Fold 3 | 22 | 188 | 15 | 8 |
| Fold 4 | 26 | 164 | 39 | 4 |
| Fold 5 | 27 | 171 | 32 | 3 |
| Reader 2, Standard | HR |  |  |  |
|  | TP | TN | FP | FN |
| Fold 1 | 146 | 20 | 27 | 40 |
| Fold 2 | 158 | 21 | 26 | 28 |
| Fold 3 | 144 | 19 | 28 | 42 |
| Fold 4 | 160 | 30 | 17 | 26 |
| Fold 5 | 157 | 33 | 14 | 29 |
| Reader 2, Standard | HER2 | | | |
|  | TP | TN | FP | FN |
| Fold 1 | 24 | 126 | 55 | 28 |
| Fold 2 | 30 | 144 | 37 | 22 |
| Fold 3 | 26 | 162 | 19 | 26 |
| Fold 4 | 33 | 152 | 29 | 19 |
| Fold 5 | 30 | 123 | 58 | 22 |
| Reader 2, Standard | Luminal | | | |
|  | TP | TN | FP | FN |
| Fold 1 | 157 | 25 | 22 | 29 |
| Fold 2 | 159 | 29 | 18 | 27 |
| Fold 3 | 162 | 31 | 16 | 24 |
| Fold 4 | 158 | 30 | 17 | 28 |
| Fold 5 | 154 | 31 | 16 | 32 |
| Reader 2, Standard | HER2-overexpressed | | | |
|  | TP | TN | FP | FN |
| Fold 1 | 8 | 168 | 48 | 9 |
| Fold 2 | 13 | 188 | 28 | 4 |
| Fold 3 | 11 | 186 | 30 | 6 |
| Fold 4 | 12 | 181 | 35 | 5 |
| Fold 5 | 12 | 159 | 44 | 5 |
| Reader 2, Standard | Triple-negative | | | |
|  | TP | TN | FP | FN |
| Fold 1 | 20 | 151 | 52 | 10 |
| Fold 2 | 21 | 156 | 47 | 9 |
| Fold 3 | 24 | 177 | 26 | 6 |
| Fold 4 | 26 | 168 | 35 | 4 |
| Fold 5 | 21 | 161 | 42 | 9 |

*TP* true positives, *TN* true negatives, *FP* false positives, *FN* false negatives.

**Supplemental references**

1. Drukker K, Anderson R, Edwards A, Papaioannou J, Pineda F, Abe H et al. (2018) Radiomics for ultrafast dynamic contrast-enhanced breast MRI in the diagnosis of breast cancer: a pilot study, vol 10575. SPIE Medical Imaging. SPIE. doi:<https://doi.org/10.1117/12.2293644>

2. Lambin P, Leijenaar RTH, Deist TM, Peerlings J, de Jong EEC, van Timmeren J et al. (2017) Radiomics: the bridge between medical imaging and personalized medicine. Nat Rev Clin Oncol 14 (12):749-762. doi:10.1038/nrclinonc.2017.141
